# Plasma Metabolomic Profiling Reveals Systemic Alterations in a Mouse Model of Type 2 Diabetes

**DOI:** 10.3390/metabo15090564

**Published:** 2025-08-22

**Authors:** Masuma Akter Brishti, Fregi Vazhappully Francis, M. Dennis Leo

**Affiliations:** Department of Pharmaceutical Sciences, College of Pharmacy, The University of Tennessee Health Science Center, 881 Madison Avenue, Memphis, TN 38163, USA; mbrisht1@uthsc.edu (M.A.B.);

**Keywords:** Type 2 diabetes, plasma metabolomics, HFD-STZ model, insulin resistance, metabolic reprogramming

## Abstract

Background: Type 2 diabetes (T2D), the most common form of diabetes, is associated with a significantly elevated risk of cardiovascular and cerebrovascular complications. However, circulating metabolic signatures that reliably predict the transition to insulin resistance, and are potentially linked to increased vascular risk, remain incompletely characterized. Rodent models, particularly those induced by a high-fat diet (HFD) combined with low-dose streptozotocin (STZ), are widely used to study the progression of T2D. However, the systemic metabolic shifts associated with this model, especially at the plasma level, are poorly defined. Methods: In this study, we performed untargeted liquid chromatography–mass spectrometry (LC-MS)-based metabolomic profiling on plasma samples from control, HFD-only (obese, insulin-sensitive), and HFD + STZ (obese, insulin-resistant) C57BL/6 mice. Results: In the HFD + STZ cohort, plasma profiles showed a global shift toward lipid classes; depletion of aromatic and branched-chain amino acids (BCAAs); accumulation of phenylalanine-derived co-metabolites, consistent with gut–liver axis dysregulation; elevations in glucose, fructose-6-phosphate, and nucleoside catabolites, indicating impaired glucose handling and heightened nucleotide turnover; increased free fatty acids, reflecting membrane remodeling and lipotoxic stress; and higher cAMP, thyroxine, hydrocortisone, and uric acid, consistent with endocrine and redox imbalance. By contrast, HFD-only mice exhibited elevations in aromatic amino acids and BCAAs relative to controls, a pattern compatible with early obesity-associated adaptation while insulin signaling remained partially preserved. KEGG analysis revealed disturbances in carbohydrate metabolism, amino acid degradation, nucleotide turnover, and hormone-related pathways, and HMDB mapping linked these changes to T2D, obesity, heart failure, and renal dysfunction. Conclusion: Collectively, these findings delineate insulin resistance-specific plasma signatures of metabolic inflexibility and inflammatory stress in the HFD + STZ model, distinguishing it from HFD alone and supporting its utility for mechanistic studies and biomarker discovery. Importantly, this plasma metabolomics study shows that insulin-sensitive and insulin-resistant states exhibit distinct variation in circulating metabolites and cardiovascular risk factors, underscoring the translational value of plasma profiling.

## 1. Introduction

Diabetes remains the seventh leading cause of death in the United States, with an estimated annual economic burden of approximately USD 250 billion [1]. Type 2 diabetes (T2D), which accounts for nearly 90% of all diabetes cases, is particularly problematic due to its multifactorial nature and chronic complications [1,2]. Vascular dysfunction in T2D is driven by a complex interplay of insulin resistance, dyslipidemia, and chronic hyperglycemia [3,4,5,6]. Consequently, there is a sustained effort among researchers and clinicians to elucidate the pathophysiological mechanisms of T2D in order to develop effective therapeutic strategies. Since lifestyle factors such as poor dietary habits and physical inactivity are central contributors to the onset of T2D, clinical interventions often emphasize lifestyle modifications, including improved nutrition and increased physical activity [7,8]. Beyond prevalence and cost, a key unmet need is the ability to distinguish obesity-related metabolic changes from those that specifically precede or accompany insulin resistance (IR), because this distinction underpins risk for macro- and microvascular complications.

Rodent models, particularly inbred mouse strains, are widely utilized to study the pathogenesis of T2D and to evaluate potential therapies [9]. Monogenic leptin/Lepr models (ob/ob, db/db) are powerful tools to investigate end-stage pathology at defined ages (e.g., nephropathy, neuropathy, vasculopathy) and for therapeutic testing in a stable, severe metabolic dysfunction background [9,10]. However, these strains are less optimal for prospectively tracking the gradual transition from insulin sensitivity to insulin resistance, because IR arises early in life and progresses rapidly from a fixed genetic lesion with a minimal insulin-sensitive baseline and limited control over timing [10,11]. More representative models have thus shifted toward diet-induced approaches that better recapitulate the gradual progression from insulin-sensitive to insulin-resistant phenotypes and overt diabetes as seen in humans [11].

The development of a nongenetic model for T2D typically involves the initial induction of insulin resistance through a high-fat diet (HFD), followed by partial β-cell dysfunction that impairs insulin secretion. This approach more closely mirrors the human disease trajectory, characterized by compensatory hyperinsulinemia, progressive β-cell decline, and eventual hyperglycemia [12]. In prior studies, we demonstrated that administration of four consecutive low doses of streptozotocin (STZ, 40 mg/kg/day/4 doses) to mice fed a 42% HFD resulted in insulin resistance and development of T2D [13,14]. A high-fat diet alone can induce insulin resistance, but it typically requires prolonged feeding, and its onset is highly variable. For example, even with a 60% fat content, insulin resistance (IR) is usually observed only after 9–11 weeks of dietary intervention [15]. Based on our observation that obese mice exhibit heightened vulnerability to β-cell injury, we developed a model combining multiple low doses of STZ with HFD feeding in C57BL/6 mice. This protocol induces an obese, hyperglycemic, insulin-resistant phenotype that models key features of human T2D; in contrast, the same STZ dose does not cause hyperglycemia in insulin-sensitive, chow-fed controls [16,17]. Critically, timed low-dose STZ after HFD yields a reproducible window for IR onset, allowing side-by-side interrogation of obesity (HFD-only) versus insulin-resistant (HFD + STZ) states in the same genetic background.

Despite the widespread use of this model, limited data exist regarding its systemic metabolic alterations, particularly at the plasma metabolome level. To fill this gap, we conducted untargeted metabolomics analysis to investigate how STZ-induced diabetes alters the metabolic pathways in this model. Given the urgent need for novel biomarkers and disease-modifying therapies, metabolomics offers a powerful platform for uncovering disease mechanisms. This rapidly evolving field within the ‘omics’ sciences enables researchers to study cellular responses to nutritional and pathological stimuli by comparing metabolic profiles between diseased and control states. Metabolomic approaches have been applied to various aspects of disease research, including biomarker discovery, drug mechanism elucidation, and identification of therapeutic targets [18,19,20,21,22].

Several studies have leveraged untargeted metabolomics in the context of T2D, identifying metabolic signatures associated with disease severity and treatment response across diverse populations [23,24,25,26]. Building on this foundation, our study was designed to investigate diet-driven changes from IR-specific alterations by directly comparing control, HFD-only (obese, insulin-sensitive), and HFD + STZ (obese, insulin-resistant) cohorts using the same platform and time-aligned sampling. In our study, we analyzed plasma from control, HFD-only, and HFD + STZ mice to identify biochemical alterations and disease-relevant metabolic pathways. We hypothesized that insulin resistance would be marked by a coordinated shift toward lipid remodeling and nucleotide turnover with depletion of specific amino acids and emergence of gut–liver co-metabolites, distinguishing it from obesity alone and providing candidate biomarker panels. This investigation aims not only to elucidate the pathophysiological underpinnings of insulin resistance and T2D but also to validate our model’s utility in reflecting the polygenic and environmentally driven nature of the human condition. By resolving IR-specific changes in circulating metabolites from obesity/hyperglycemia alone-related signals, this work lays the groundwork for circulating biomarkers to guide the prevention and treatment of diabetic vascular disease. Ultimately, our findings may shed light on how insulin resistance contributes to vascular complications such as stroke, myocardial infarction, hypertension, and peripheral artery disease, thereby informing future research into targeted interventions.

## 2. Materials and Methods

### 2.1. Animal Usage

All animal procedures were conducted following institutional guidelines and were approved by the Institutional Animal Care and Use Committee (IACUC) at the University of Tennessee Health Science Center (UTHSC). Male C57BL/6J mice were used for all experiments. A type 2 diabetes model was established using a high-fat diet (HFD) combined with low-dose streptozotocin (STZ) and has been previously validated by our group [13,14]. Briefly, at 6 weeks of age, mice were initiated on an HFD (TD.88137, Inotiv, Chicago, IL, USA). After eight weeks on the HFD, mice received intraperitoneal injections of STZ (Sigma Aldrich, St. Louis, MO, USA) at a dose of 40 mg/kg/day for four consecutive days to induce insulin resistance. The mice continued the HFD for an additional two weeks following STZ administration. Mice were euthanized either before or after STZ treatment, depending on the experimental requirement. Euthanasia was performed using 1.5% isoflurane anesthesia followed by decapitation. Blood samples were collected and centrifuged at 2000 rpm for 10 min to isolate plasma from the supernatant. Blood glucose was assessed using AlphaTrak2 glucose test strips, and plasma insulin was evaluated using a mouse insulin ELISA kit (Crystal Chem, Elk Grove Village, IL, USA). The experimental groups included regular chow controls, P-treatment group (HFD only, without STZ), and the T-treatment group (HFD with STZ). A total of 27 mice, comprising 9 mice from each group, were used. Samples were pooled into groups of 3 for metabolomic analysis. A separate cohort of 5 mice in each group was used for glucose and insulin tolerance tests. Oral glucose tolerance test (GTT) was performed as previously described and summarized as AUC [27]. Protocol for the insulin tolerance test (ITT) followed previously described standard methodology [27]. The homeostasis model assessment-2 (HOMA2-IR) index and insulin sensitivity index was calculated using the calculator available on the Diabetes Trials Unit of the University of Oxford website (https://www.dtu.ox.ac.uk/homacalculator/; accessed: 28 September 2023) [28].

### 2.2. Plasma Metabolomic Analysis Using UPLC-MS/MS

Untargeted metabolomic profiling was performed using ultra-performance liquid chromatography–tandem mass spectrometry (UPLC-MS/MS) to identify and quantify metabolites with biological relevance. Instrumentation: all metabolomic analyses were carried out using the following equipment: Mass Spectrometer TripleTOF 6600+ (SCIEX, Redwood City, CA, USA) and UHPLC System (ExionLC AD, SCIEX, Redwood City, CA, USA).

### 2.3. Sample Preparation

Plasma samples stored at −80 °C were thawed on ice and briefly vortexed (10 s). For each sample, 50 μL of plasma was mixed with 300 μL of extraction solvent (acetonitrile/methanol, 1:4 *v*/*v*) containing internal standards in a 2 mL microcentrifuge tube. Samples were vortexed for 3 min and centrifuged at 12,000 rpm for 10 minat 4 °C. The resulting supernatant (200 μL) was transferred to a fresh tube and incubated at −20 °C for 30 min, followed by a second centrifugation (12,000 rpm, 3 min, 4 °C). A 180 μL aliquot of the clarified supernatant was used for LC-MS analysis.

### 2.4. Chromatographic Conditions

The conditions for hydrophilic interaction chromatography (HILIC) were as follows: Column: ACQUITY Premier BEH Amide (1.7 μm, 2.1 mm × 150 mm); Mobile Phase A: 60% acetonitrile, 30% water, 10% methanol with 20 mM ammonium formate (pH of 10.6); Mobile Phase B: 40% acetonitrile, 60% water with 20 mM ammonium formate (pH of 10.6); Flow Rate: 0.40 mL/min; Column Temperature: 40 °C; Injection Volume: 4 μL. 

### 2.5. Data Preprocessing and Metabolite Identification

Raw LC-MS data files were converted to the mzXML format using ProteoWizard v3.0. Peak detection, alignment, and retention time correction were performed using the XCMS software package. Peaks with a missing value rate >50% within any group were excluded. Missing values were imputed using the K-nearest neighbor (KNN) algorithm, and signal intensities were normalized using support vector regression (SVR). Metabolite identification was performed by integrating multiple resources, including a proprietary in-house database, public repositories, predictive libraries, and the metDNA platform. Only compounds with a comprehensive identification score ≥0.7 and coefficient of variation (CV) <0.3 across quality control (QC) samples were retained. Data from positive and negative ionization modes were combined, retaining the feature with the highest identification confidence and lowest CV.

### 2.6. Data Quality Evaluation

**Figure 1:**
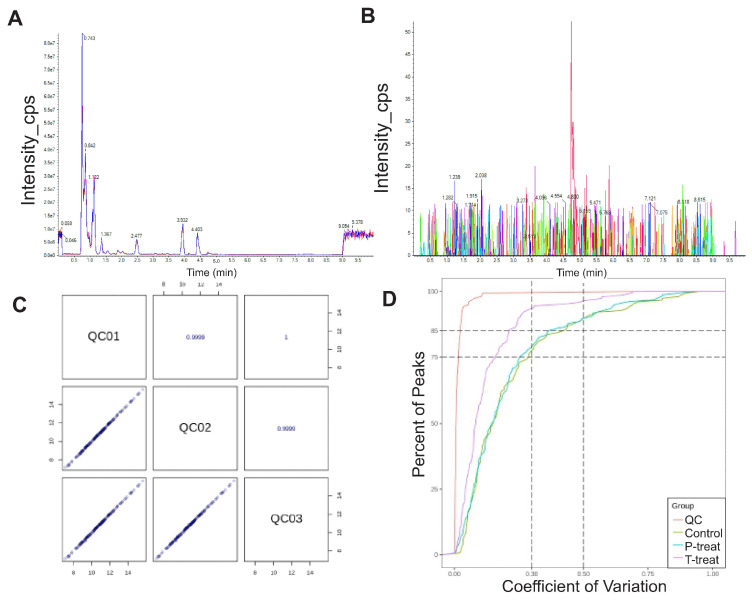
(**A**) TIC overlap diagram detected by QC sample essence spectrum. (**B**) EIC diagram of the internal label in the blank sample. The signals in the above EIC graphs are all noise peaks, and the internal standard substance has no prominent signal peaks at the corresponding time. (**C**) Plot of QC sample correlation. The bottom left square of the diagonal line is the correlation scatter plot of the corresponding QC samples. The horizontal and vertical coordinates represent the metabolite content (for Log processing), and each point in the plot corresponds to a single metabolite. The upper right square of the diagonal line is the Pearson correlation coefficient of the corresponding QC samples. (**D**) CV distribution of each group.

Quality Control (QC) Sample Monitoring: A pooled QC sample, created by combining aliquots from all experimental samples, was analyzed periodically (1 QC per 10 test samples) to monitor instrument stability and reproducibility. Total Ion Chromatograms (TICs): Overlay of TICs from QC samples demonstrated high reproducibility and stability across the LC-MS runs (Figure 1A). Blank Sample Analysis: Blank samples were included intermittently to assess background noise and contamination. No significant peaks corresponding to internal standards were detected, confirming minimal carryover (Figure 1B). QC Correlation Analysis: Pearson’s correlation coefficients (r) were calculated among QC replicates. High correlation values (|r| ≈ 1) indicated robust instrument performance and consistent sample preparation. Internal Standard Consistency: Internal standards spiked into all QC samples exhibited minimal variation (CV ≤ 15%), further validating analytical stability and data reliability (Figure 1C). Coefficient of Variation (CV) Distribution: The empirical cumulative distribution function (ECDF) was used to evaluate CV values across all metabolites. Over 85% of the features detected in QC samples showed CV < 0.5, and more than 75% had CV < 0.3, indicating excellent reproducibility and low technical variability (Figure 1D).

### 2.7. Statistical Analysis

In the main figures and Supplementary Materials, P indicates the HFD-only group (no STZ) and T indicates the HFD + STZ group. All data underlying the results are provided in the supplements. Statistical analyses were performed in R (v4.4.1) and MetaboAnalyst 5.0. Prior to analysis, feature intensities were log-transformed and Pareto-scaled. Principal component analysis (PCA) and orthogonal partial least squares discriminant analysis (OPLS-DA) were used to visualize group structure and identify discriminative features; variable importance in projection (VIP) scores from OPLS-DA ranked metabolites by their contribution to separation (VIP ≥ 1 denotes above-average influence on the model). For univariate testing, data normality was assessed on model residuals (Shapiro–Wilk) and by Q-Q plots. When assumptions were met, we applied unpaired *t*-tests or one-way ANOVA with appropriate post-hoc comparisons, and when not met, we used the Mann–Whitney U test. Multiple testing was controlled using Benjamini–Hochberg false discovery rate (q < 0.05). Metabolites were considered differentially abundant if they met |log_2_ fold change| ≥ 1, VIP ≥ 1, and FDR-adjusted q < 0.05, prioritizing biologically meaningful effect sizes. Pathway enrichment and functional annotation were performed using KEGG (over-representation analysis) and HMDB mapping. Figures (volcano, heatmaps, VIP plots) were generated in R (ggplot2, pheatmap) with consistent thresholds and annotations indicated in captions.

## 3. Results

### 3.1. Low-Dose Streptozotocin Reliably Induces Insulin-Resistance in High-Fat Diet-Fed Mice

**Figure 2:**
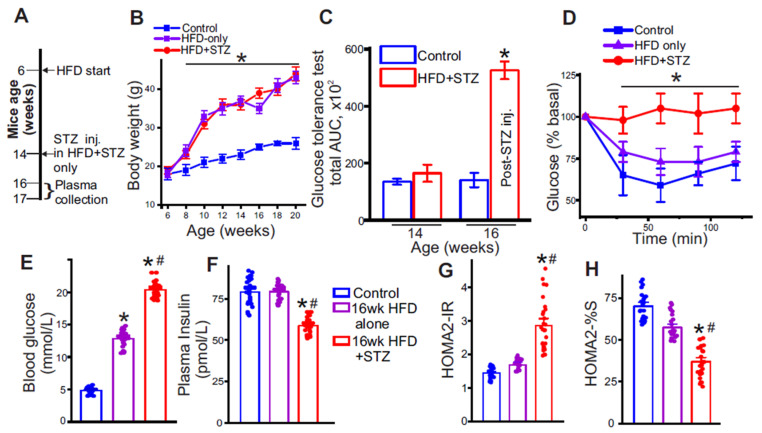
Induction of T2D in mice using a high-fat diet (HFD) + low-dose streptozotocin protocol. (**A**) Methodology schematic of the diet/STZ protocol followed. (**B**) Body weight (in g) recordings from 6 weeks of age to 20 weeks of age in different groups. n = 30 for each. * *p* < 0.05 vs. control. (**C**) Glucose tolerance test (GTT) in control and in HFD mice before (week 14) and after (week 16) STZ injections. n = 5 each, * *p* < 0.05 vs. control. (**D**) Insulin tolerance test (ITT) performed at week 16 in different groups. n = 5 each, in the HFD + STZ group, * *p* < 0.05 vs. control. (**E**) Fasting blood glucose (in mmol/L), (**F**) Plasma insulin in different groups, (**G**) HOMA2-IR and (**H**) insulin sensitivity (HOMA2-%S) indices of all mice recorded at 16 weeks of age. n = 27 each, * *p* < 0.05 vs. control and ^#^ *p* < 0.05 vs. HFD only.

Type 2 diabetes was induced using the HFD–low-dose STZ regimen (Figure 2A). Treatment animals showed steady weight gain on HFD, reaching ~40 g after 8 weeks of feeding (Figure 2B). Over the same period, fasting glycemia rose to ~12.5 mmol/L-about 2.3-fold above controls (~5.5 mmol/L) (Figure 2E). After STZ administration, fasting glucose increased further to ~20 mmol/L (~3.6-fold vs. control) (Figure 2E). Glucose tolerance tests showed a mild upward shift after 8 weeks of HFD. In contrast, a clear, significant impairment emerged only following STZ injections (Figure 2C). Insulin tolerance test (ITT) results demonstrated impaired insulin responsiveness in HFD + STZ group versus HFD-only, as evidenced by a diminished decline in blood glucose (Figure 2D). Further, insulin resistance was also estimated with the HOMA2-IR calculator [29] using fasting glucose (Figure 2E) and plasma insulin (Figure 2F); values were ~1.8 after 10 weeks on HFD alone compared to ~3 post-STZ injections (Figure 2G). Insulin sensitivity index proportionally decreased during the same period to ~35% in the HFD + STZ group compared to ~60% in the HFD-only group. Thus, the HFD-only group was obese and hyperglycemic, but insulin sensitive. The HFD + STZ group was obese, hyperglycemic, and insulin resistant. Collectively, these results indicate that the HFD + STZ protocol produced obesity, fasting hyperglycemia, and insulin resistance consistent with a T2D-like phenotype in mice.

### 3.2. Metabolite Class Distribution and PCA Highlight Systemic Metabolic Remodeling

**Figure 3:**
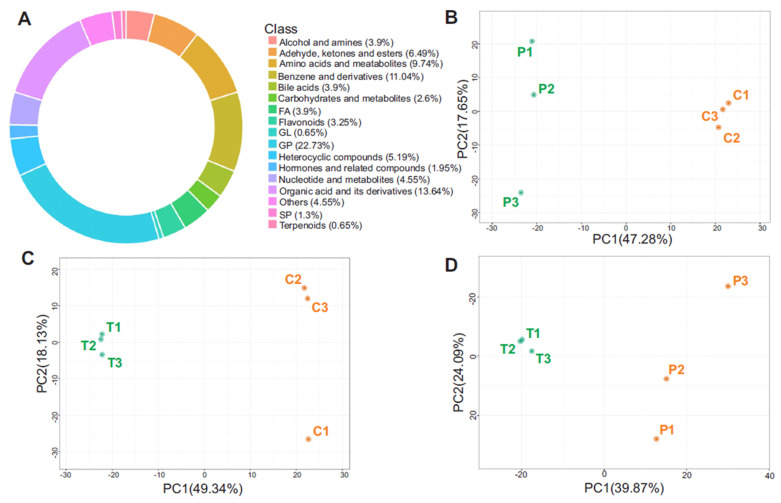
(**A**) Ring diagram of metabolite classes. The area of each color block represents the proportion of the category. Principal component analysis of different groups. (**B**) P treatment vs. control, (**C**) T treatment vs. control, and (**D**) T treatment vs. P treatment. Each dot in the figure represents a sample; the same color represents samples in the same group, and the group is a grouping. Note: C is untreated control, P-treatment is HFD only, T-treatment is HFD + STZ.

To gain an initial understanding of metabolic alterations across treatment groups, we performed metabolite composition ratio analysis to evaluate the relative distribution of major metabolite classes in plasma samples. This approach provides a global view of the biochemical landscape, allowing for the visualization of how different treatment conditions shift the balance among key metabolite types, such as lipids, amino acids, nucleotides, carbohydrates, and others. As shown in Figure 3A, the ring diagram illustrates the proportion of metabolite classes across all samples. Principal component analysis (PCA) was performed on all plasma metabolomics data, including QC samples, to assess overall clustering patterns and variability between groups. PCA is an unsupervised dimensionality-reduction method that transforms thousands of correlated metabolite features into a few orthogonal principal components that capture the greatest variance. It is used to visualize the overall data structure, revealing natural sample clusters, trends, and potential outliers, without imposing group labels. Here, PCA serves as an unbiased quality and separation check (tight QC clustering; variance explained by PC axes) and quantifies how much variance distinguishes the control, HFD, and HFD + STZ groups, thereby justifying subsequent differential and pathway analyses. The score plots revealed clear separation between treatment groups, indicating significant differences in the global metabolic profiles. In Figure 3B, the HFD-only group showed distinct separation from the control group along the X-axis, reflecting significant differences in metabolic composition. Figure 3C demonstrated a similar separation between the HFD + STZ group and the control, while Figure 3D revealed distinct metabolic shifts between the STZ-treated group and the HFD-only group. These PCA results support the existence of robust group-specific metabolic alterations.

### 3.3. Orthogonal Partial Least Squares Discriminant Analysis (OPLS-DA)

**Figure 4:**
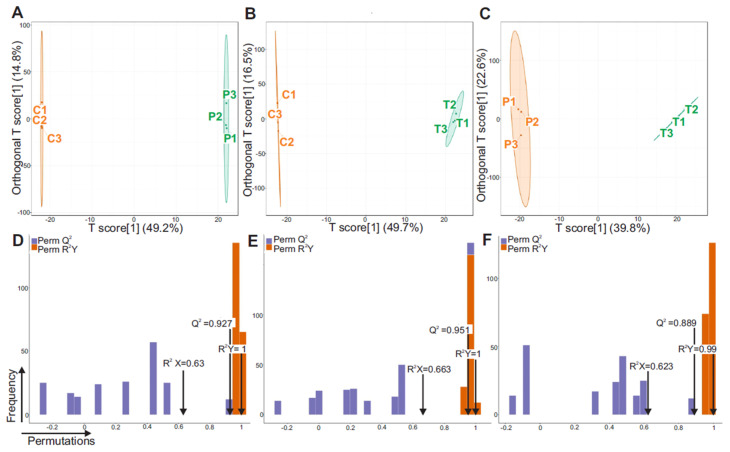
OPLS-DA score diagram. (**A**) P treatment vs. control, (**B**) T treatment vs. control, (**C**) T treatment vs. P treatment. The X-axis shows between-group variation (predicted component), while the Y-axis reflects within-group variation (orthogonal component). OPLS-DA validation plots for (**D**) P treatment vs. control, (**E**) T treatment vs. control, (**F**) T treatment vs. P treatment. The horizontal coY-axis indicates the model R^2^Y, Q^2^ values, and the vertical coY-axis is the frequency of the model classification effect. Note: C is untreated control, P-treatment is HFD only, T-treatment is HFD + STZ.

To further refine group separation and identify key discriminatory metabolites, Orthogonal Partial Least Squares Discriminant Analysis (OPLS-DA) was employed. Unlike PCA, OPLS-DA is a supervised approach that enhances the ability to detect class-related metabolic changes by partitioning the X matrix into components that are predictive of group differences and orthogonal (unrelated) variation. This method improves interpretability by isolating the variation relevant to treatment effects, thereby enhancing biomarker discovery. OPLS-DA score plots demonstrated clear discrimination among the control, HFD-only, and HFD + STZ groups (Figure 4A–C, Supplement S1–S3), consistent with the trends observed in PCA. The robustness and predictive accuracy of each OPLS-DA model were validated using permutation testing (Figure 4D–F). These permutation plots showed that the original model’s R^2^Y and Q^2^ values were significantly higher than those of the permuted models, confirming that the observed class separations were not due to overfitting or random chance. The consistent clustering and high model validity support the presence of distinct and reproducible metabolic alterations induced by each treatment condition.

### 3.4. Volcano Plot of Differential Metabolites

**Figure 5:**
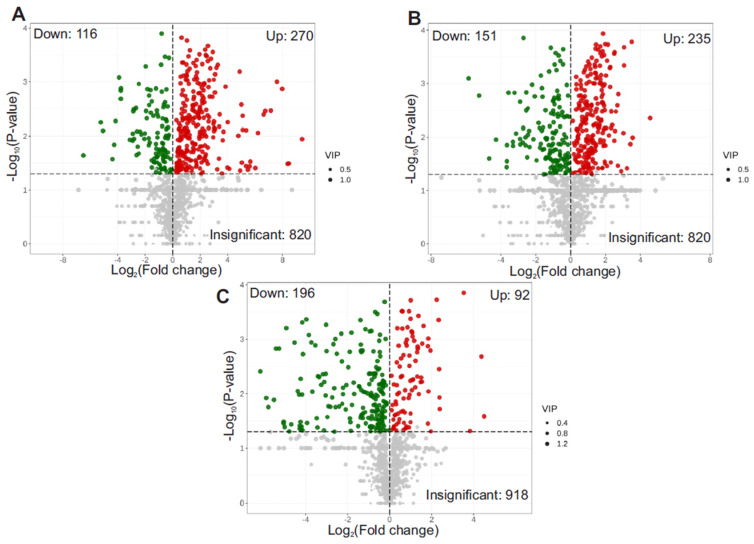
Volcano plot of differential metabolites for (**A**) P treatment vs. control, (**B**) T treatment vs. control, (**C**) T treatment vs. P treatment. The X-axis represents the (log_2_FC) value of metabolites between two groups. Y-axis represents the level of significant differences (−log_10_P-value). The size of each dot indicates VIP value. Note: C is untreated control, P-treatment is HFD only, T-treatment is HFD + STZ.

Volcano plots were used to illustrate the fold changes and statistical significance of differential metabolites between treatment groups. Each point represents a metabolite, with red indicating upregulation, green indicating downregulation, and gray indicating no significant change. The X-axis shows the log_2_ fold change (log_2_FC), and the Y-axis shows the −log_10_ *p*-value. These plots were further refined using the variable importance in projection (VIP) scores from the OPLS-DA model. VIP scores reflect the relative contribution of each metabolite to group separation in the multivariate model. Point size in the volcano plots corresponds to the VIP score. Accordingly, in the P treatment vs. control, 270 metabolites were significantly upregulated, and 116 were downregulated. T treatment vs. control: 225 metabolites were significantly upregulated, and 151 were downregulated. T treatment vs. P treatment: 92 metabolites were significantly upregulated, and 196 were downregulated (Figure 5A–C, Supplement S4–S6). The top 25 DEGs in the HFD + STZ group compared to the HFD-only group are shown in Table 1.

### 3.5. Heatmap of Differential Metabolite Abundance

**Figure 6:**
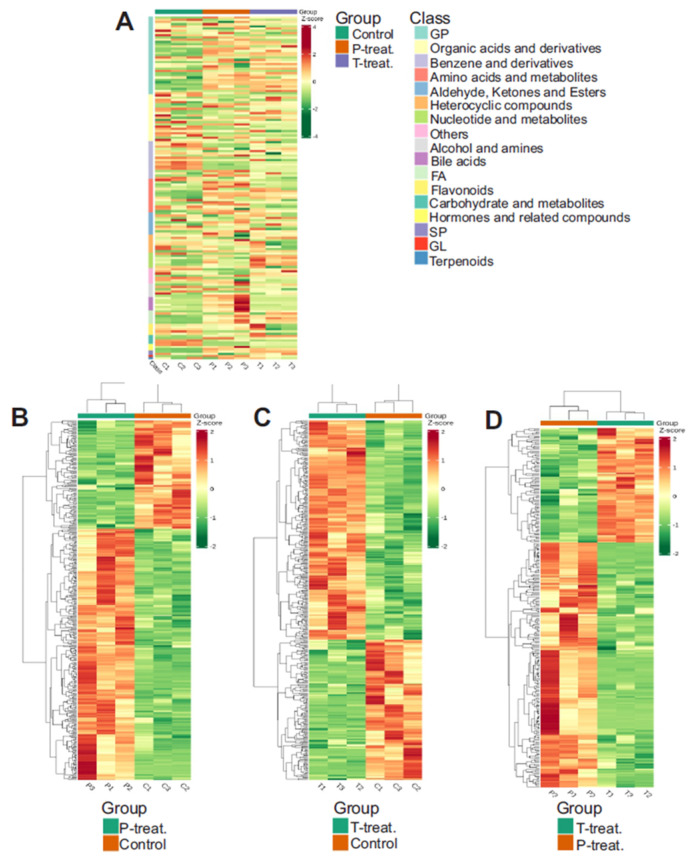
(**A**) Heatmap with all samples showing different classes of metabolites. Clustered heatmaps for (**B**) P treatment vs. control, (**C**) T treatment vs. control, (**D**) T treatment vs. P treatment. Note: C is untreated control, P-treatment is HFD only, T-treatment is HFD + STZ.

To visualize patterns in metabolite abundance across samples, a heatmap was generated using unit variance scaling to normalize the data. This enabled comparison of relative fold changes in differential metabolites across treatment groups. Figure 6A presents an unclustered heatmap displaying all samples and their corresponding metabolite classes. Clustered heatmaps comparing group-specific metabolite profiles are shown in Figure 6B–D. 

### 3.6. KEGG Pathway Enrichment

**Figure 7:**
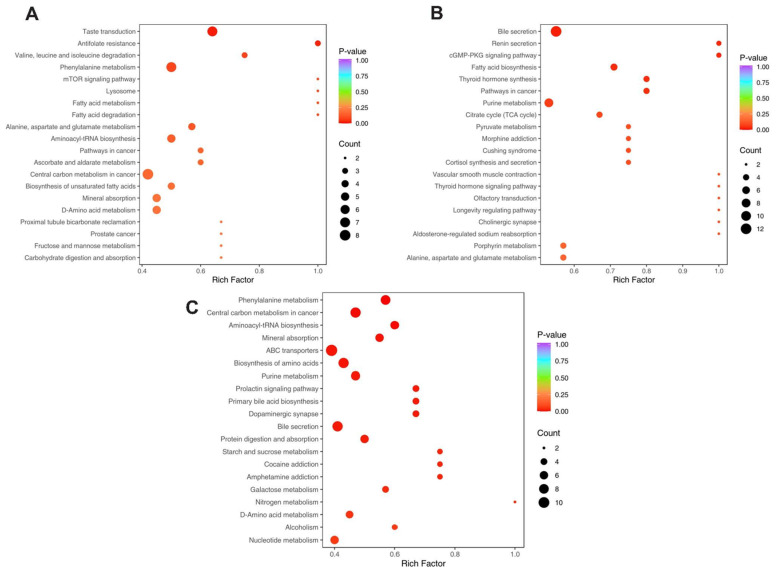
KEGG pathway enrichment analysis of differential metabolites. (**A**) P treatment vs. control, (**B**) T treatment vs. control, (**C**) T treatment vs. P treatment. The X-axis indicates the Rich Factor (ratio of differential to total metabolites in a pathway), while the Y-axis lists the KEGG pathways. Dot color reflects statistical significance (*p*-value), with darker red indicating greater significance. Dot size corresponds to the number of enriched differential metabolites in each pathway. Note: C is untreated control, P-treatment is HFD only, T-treatment is HFD + STZ.

Functional enrichment analysis was performed using the Kyoto Encyclopedia of Genes and Genomes (KEGG) database to map significantly altered metabolites to biological pathways. Enrichment was quantified using the Rich Factor, defined as the ratio of the number of differential metabolites involved in a given pathway to the total number of metabolites annotated in that pathway. A higher Rich Factor indicates a greater degree of enrichment. Significant pathways were identified and grouped by the extent of metabolite involvement. The results are visualized in Figure 7A–C and detailed in Supplement S7–S9, which categorizes pathways into two tiers, more affected and less affected, based on the number of differentially enriched metabolites. All listed pathways showed statistically significant enrichment.

### 3.7. Pathway-Level Clustering Reveals Key Metabolic Disruptions in Diabetes

**Figure 8:**
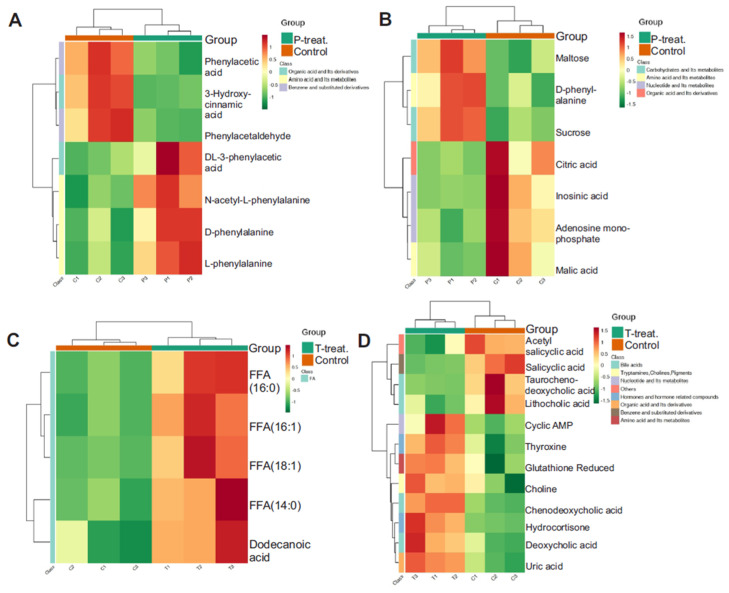
Clustering heat map of differential metabolites in KEGG pathway for P treatment vs. control (**A**,**B**) and T treatment vs. control (**C**,**D**). Note: C is untreated control, P-treatment is HFD only, T-treatment is HFD + STZ.

**Figure 9:**
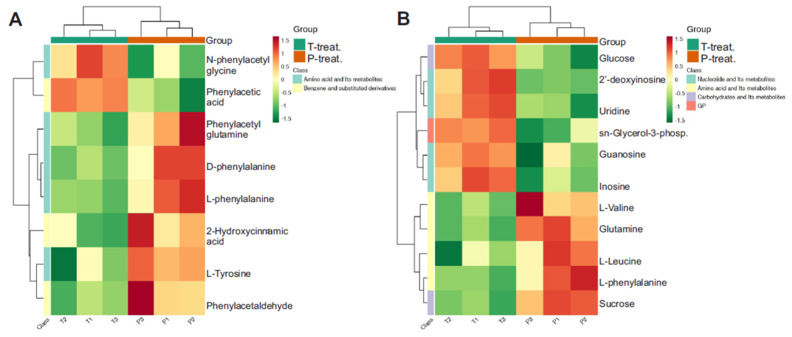
Clustering heat map of differential metabolites in KEGG pathway for T treatment vs. P treatment (**A**,**B**). Note: C is untreated control, P-treatment is HFD only, T-treatment is HFD + STZ.

To further explore the biological relevance of the observed metabolic alterations, five significantly enriched KEGG metabolic pathways were selected for clustering analysis. These pathways represent the most impacted biochemical routes in the dataset and were chosen based on their statistical significance and pathway coverage. Only pathways containing five or more differentially expressed metabolites were included. These results revealed several interesting observations. When compared to the control, the HFD only group showed an increase in L-phenylalanine, D-phenylalanine, and N-acetyl-L-phenylalanine along with an increase in maltose and sucrose, but a decrease in citric acid, malic acid, and adenosine monophosphate (AMP) (Figure 8A,B). In contrast, when compared with controls, the major metabolite pathways altered in the HFD + STZ group were several free fatty acids (FFAs) and dodecanoic acid, which were increased (Figure 8C). Several other metabolites were also increased in the HFD + STZ group compared to controls, including cyclic AMP, thyroxine, choline, hydrocortisone, deoxycholic acid, and uric acid (Figure 8D). Interestingly, when the HFD group was compared with the HFD + STZ group, several notable differences emerged. For example, L-phenylalanine, D-phenylalanine, and L-tyrosine levels were lower, but phenylacetic acid and N-phenylacetyl glycine levels were higher in the HFD + STZ group (Figure 9A). Similarly, glucose, 2’-deoxyinosine, uridine, and inosine levels were elevated in the HFD + STZ group compared to HFD only (Figure 9B). These results suggest a remarkable metabolic shift occurring between the HFD only, obese, non-insulin resistant group and the HFD + STZ, obese, insulin-resistant group.

### 3.8. HMDB-Based Disease and Pathway Annotation

To further contextualize the differential metabolites, we performed annotation using the Human Metabolome Database (HMDB). This analysis focused on primary metabolic pathways and their associated disease annotations. The HMDB platform links metabolite data with over 40,000 endogenous compounds and associated protein/gene information, incorporating references to external databases such as KEGG, Metlin, and Biocyc. Disease associations were mapped for key metabolites based on annotations from the HMDB. A detailed list of diseases for each comparative group is provided in the Supplement (S10–S12). These data provide insight into the potential clinical relevance of the observed metabolic alterations. These findings indicate distinct metabolite profiles in each group, suggesting that STZ treatment induces broader metabolic alterations beyond those caused by the high-fat diet alone.

## 4. Discussion

This untargeted plasma metabolomics study reveals distinct metabolic profiles between high-fat diet (HFD)-fed mice and those exposed to both HFD and low-dose streptozotocin (STZ). The metabolic alterations observed reflect progressive pathophysiological transitions from diet-induced obesity to insulin-resistant, diabetic states. Notably, while both HFD and HFD + STZ groups exhibit obesity-related metabolic perturbations, the HFD + STZ group displays broader and more severe disruptions, consistent with an insulin-resistant phenotype involving systemic inflammation, impaired glucose handling, and altered gut–liver axis communication. 

We modeled insulin resistance in C57BL/6 mice using a high-fat diet combined with low-dose streptozotocin (HFD + STZ). This inducible model was chosen so that the onset of insulin resistance could be precisely aligned with experimental readouts and because the HFD + STZ approach captures key polygenic features of human type 2 diabetes [11,29,30,31]. While HFD feeding alone has been used to achieve insulin resistance, this model is susceptible to diet composition. In many reports with HFD alone, mice are fed ~60% kcal from fat, yet reliable insulin resistance typically emerges only after ~9–11 weeks on the diet [15,32]. Given that U.S. dietary fat intake is closer to 30–40% of energy [33,34], we used the TD.88137 ‘Western diet’, which provides ~45% kcal from milk fat. With diet alone, we observed that mice generally reached HOMA2-IR ~2 or higher after 10–14 weeks. This broad range in onset times prevented us from prospectively identifying when insulin resistance would emerge in any given cohort. In contrast, administering low-dose STZ after ~8 weeks of HFD feeding produced a consistent insulin-resistant phenotype, with HOMA2-IR values >2. Following the establishment of this T2D model, we then performed untargeted plasma metabolomics. Our goal with using this approach in both HFD-only and HFD + STZ cohorts was to investigate obesity-driven dysfunction from STZ-mediated β-cell injury and to resolve insulin resistance-specific circulating signatures and pathway shifts that have significant translational relevance.

The global metabolite composition analysis showed a shift in major metabolite classes, with the HFD + STZ group demonstrating increased proportions of lipids and a reduction in amino acids and nucleotides, reflecting a transition toward metabolic inflexibility, impaired substrate switching, and an increased reliance on lipid oxidation at the expense of carbohydrate utilization. PCA and clustering further confirmed these group-specific profiles, highlighting that STZ induces a distinct diabetic metabolic state beyond that caused by HFD alone.

A distinct pattern of amino acid regulation emerged when comparing the HFD-only group (obese, diabetic, insulin-sensitive) to the HFD + STZ group (obese, diabetic, insulin-resistant). In the HFD-only group, levels of several aromatic amino acids, L-phenylalanine, D-phenylalanine, and N-acetyl-L-phenylalanine, were significantly elevated compared to controls. This accumulation is consistent with early metabolic disturbances related to obesity and low-grade inflammation, where reduced amino acid clearance and altered hepatic enzyme activity accompany adipose tissue expansion, yet insulin signaling remains partially preserved [35,36]. 

Similarly, levels of branched-chain amino acids (BCAAs; leucine, isoleucine, and valine) were elevated in the HFD-only group compared to the HFD + STZ group. In obesity, elevated BCAAs may arise from reduced skeletal muscle oxidation and incomplete catabolism, contributing to mTOR activation and insulin resistance; however, in overt diabetes, enhanced proteolysis and altered gut microbiota activity can accelerate their depletion. This reduction in BCAAs in the insulin-resistant state suggests a shift from amino acid accumulation, typical of early obesity-related metabolic stress, toward enhanced catabolism and metabolic reprogramming in overt diabetes. Such changes may reflect increased hepatic nitrogen disposal via the urea cycle, altered anaplerotic flux into the TCA cycle, and intensified host–microbiota co-metabolism of amino acids under chronic hyperglycemia. These findings are consistent with previous studies demonstrating the complex role of BCAAs in diabetes pathogenesis and their predictive value as biomarkers of insulin resistance [37,38,39]. 

The concurrent elevation of glucose, fructose-6-phosphate, and purine metabolites (e.g., inosine, 2′-deoxyinosine, uridine) in the HFD + STZ group further supports the presence of metabolic inflexibility and systemic stress. These metabolites also point toward increased nucleotide turnover and DNA/RNA breakdown, possibly linked to oxidative damage and heightened cell turnover following insulin resistance. Additionally, these results align with prior reports showing that prolonged high-fat feeding alone can induce insulin resistance, and that low-dose STZ effectively accelerates this transition toward a more pronounced insulin-resistant and diabetic phenotype.

In contrast, the HFD + STZ group showed a reduction in these same amino acids (L-phenylalanine, D-phenylalanine, and L-tyrosine) compared to the HFD-only group, alongside an increase in phenylacetic acid (PAA) and N-phenylacetyl glycine (PAGly), microbial [40] and hepatic conjugation products of phenylalanine metabolism [41]. This aromatic amino acid shift is a recognized signature of gut dysbiosis in insulin resistance, where phenylalanine is increasingly shunted toward microbial fermentation and hepatic detoxification, generating uremic toxins with vascular effects. These metabolites are recognized markers of gut–liver axis dysregulation and have been linked to cardiometabolic risk, endothelial dysfunction, and renal stress in both humans and rodent models [40,41,42].

Together, these findings demonstrate that amino acid metabolism is not uniformly elevated in diabetes but instead reflects the stage and severity of metabolic disruption. The HFD-only group retains a phenotype of metabolic adaptation. In contrast, the HFD + STZ group exhibits a more catabolic, insulin-resistant profile, characterized by amino acid depletion and diversion into downstream pathways that involve microbial fermentation and hepatic clearance.

Carbohydrate-related metabolites also demonstrated divergent trends between groups. The HFD-only mice showed increased levels of maltose and sucrose, reflecting altered carbohydrate digestion or absorption. In contrast, the HFD + STZ group exhibited elevated levels of glucose, D-fructose-6-phosphate, and uridine, consistent with impaired peripheral glucose uptake, hepatic gluconeogenesis, and increased nucleotide salvage pathway activity [43,44]. Elevated citric acid and malic acid in the HFD-only group suggest preserved mitochondrial TCA cycle activity, possibly as a compensatory response to overnutrition. However, the reduction in citric acid and AMP in the HFD group compared to controls may also signal early energy stress or reduced oxidative capacity [45,46,47]. The further disruption of TCA intermediates and nucleotide phosphates in the HFD + STZ group is consistent with mitochondrial dysfunction, reduced oxidative phosphorylation efficiency, and a shift toward anaerobic metabolism.

The HFD + STZ group exhibited a pronounced increase in circulating free fatty acids (FFAs), including palmitic, oleic, palmitoleic, myristic, and dodecanoic acids, compared to both controls and HFD-only mice. These saturated and monounsaturated FFAs are potent inducers of ER stress, mitochondrial ROS generation, and inflammatory signaling via TLR4 and NF-κB pathways [48]. Elevated phosphatidylcholine and phosphatidylethanolamine were also observed in this group and further reflect membrane remodeling and lipid mobilization, consistent with insulin resistance and lipotoxicity [49].

The HFD + STZ group displayed a broad endocrine and redox imbalance. Levels of cyclic AMP (cAMP), thyroxine (T4), and hydrocortisone were significantly increased, reflecting hormonal dysregulation that promotes gluconeogenesis, lipolysis, and inflammation [50,51,52]. Thyroxine excess in this context may reflect altered thyroid hormone metabolism under systemic inflammation, while elevated glucocorticoids are a hallmark of chronic stress signaling in metabolic disease. Elevated uric acid levels in this group suggest increased purine catabolism and oxidative stress, which may further exacerbate endothelial dysfunction and increase cardiovascular risk [53].

These findings collectively reveal a metabolic divergence between HFD-only mice, which demonstrate signs of early metabolic adaptation, and HFD + STZ mice, which exhibit a phenotype of systemic insulin resistance and metabolic collapse. The former group exhibits elevated levels of amino acids and sugars, with no significant evidence of impaired energy metabolism or inflammatory stress. In contrast, the latter displays amino acid depletion, lipid accumulation, microbial metabolite buildup, and hormonal derangement, indicating a transition to a metabolically inflexible, insulin-resistant state.

Limitations: 1. Although we have experience in using diabetic female mice, the present study used male C57BL/6 mice to allow better interpretability of the results. Female C57BL/6 mice show relative resistance to HFD-alone or HFD + STZ-induced diabetes [31] (~30% progress to overt T2D under comparable conditions), and estrous-related hormonal fluctuations introduce an additional variance that can confound primary metabolic readouts. We therefore restricted this cohort to males and will conduct a dedicated study in females with estrous tracking to investigate T2D-mediated changes across cycle stages in more detail. 2. We focused on untargeted plasma metabolomics to obtain an integrative, translatable snapshot of whole-body metabolism at key pre- and post-STZ inflection points, allowing us to investigate whether circulating signatures can flag emerging insulin resistance. More detailed tissue-resolved and flux analyses were beyond the scope of the present study and are planned for follow-up studies.

## 5. Conclusions

These data demonstrate that the HFD + STZ model of type 2 diabetes elicits broad and biologically significant alterations across multiple metabolic domains, including disrupted amino acid homeostasis, impaired carbohydrate and energy metabolism, increased lipid mobilization, nucleotide imbalance, and hormonal dysregulation. The resulting metabolomic profile closely recapitulates key features of insulin-resistant diabetes in humans. These results highlight the highly dynamic and stage-specific nature of metabolic remodeling during diabetes progression, underscoring the importance of phenotypic characterization in animal models for investigating disease mechanisms and evaluating therapeutic strategies.

## Figures and Tables

**Table 1:** Top up- or downregulated DEGs by fold change in the HFD + STZ group vs. HFD-only. Note: possible contaminants or artifacts are not listed.

	Upregulated	Downregulated
1	[(2R)-1-[(Z)-hexadec-9-enoyl]oxy-3-phosphonooxypropan-2-yl] tetracosanoate	N-[(3a,5b,7b)-7-hydroxy-24-oxo-3-(sulfooxy)cholan-24-yl]-Glycine
2	14,15-Leukotriene C4(ExC4)	Gln-Leu-Glu-Lys
3	Inosinic acid	Phe-Leu-Gln-Lys
4	Trp-Ser-Ala	His-Val-Thr-Glu-Glu
5	PE-NMe(16:1(9Z)/20:1(11Z))	Tyr-Gln-Thr-Lys
6	Linoleylethanolamide	Leu-Tyr-Asp-Lys
7	Aldehydo-D-Galactose	Leu-Ser-Ala-Leu-Glu
8	3-Sulfocatechol	Val-Asp-Ile-Arg
9	Symmetric dimethylarginine	Lys-Gln-Ile-Glu
10	Glucose 1-phosphate	Thr-Val-Leu-Thr-Ser
11	D-Fructose-6-phosphate	Arg-Thr-Ile-Glu
12	Inosine	Ser-Phe-Val-Lys
13	3-Hydroxybutyric acid	Asn-Lys-Arg-Asp
14	Glucose	Tyr-Gln-Asn-Glu
15	Isobutyrylglycine	Taurohyodeoxycholic acid
16	2′-Deoxyinosine	Ile-Phe-Gln-Glu
17	5-phospho-alpha-D-ribose cyclic-1,2-phosphate	Tauroursodeoxycholic acid
18	1H-Indole-3-acetaldehyde, 5-methoxy-	Tyr-Glu-Val-Lys
19	(9Z)-N-[2-(5-hydroxy-1H-indol-3-yl)ethyl]octadec-9-enamide	Glu-Ser-Val-Pro-Glu
20	Sedoheptulose	Leu-Ala-Gly-Glu-Phe
21	Aspartyl alanine	Ser-His-Glu-Ala-Glu
22	Allantoic acid	Glu-Pro-Gly-Tyr-Ser
23	Arabinose-5-phosphate	Val-Tyr-Ser-Lys
24	Val-Phe-Lys	Taurochenodeoxycholic acid
25	Arachidonoyl Serotonin	Arg-Gln-Ser-Lys

## Data Availability

All data used to derive the results and figures in this manuscript are provided as Supplementary Materials S1–S12.

## Supplementary Materials

All supplemental files (S1–S12) listed in the manuscript can be downloaded at: https://doi.org/10.5281/zenodo.16112775.

## Abbreviations

The following abbreviations are used in this manuscript:

FA	Fatty Acid
T2D	Type 2 Diabetes
HFD	High-Fat Diet
STZ	Streptozotocin
IR	Insulin Resistance
LCMS	Liquid Chromatography Mass Spectrometry
